# *In vivo* validation of psilacetin as a prodrug yielding modestly lower peripheral psilocin exposure than psilocybin

**DOI:** 10.3389/fpsyt.2023.1303365

**Published:** 2024-01-08

**Authors:** Nathan T. Jones, Laura Wagner, Molly C. Pellitteri Hahn, Cameron O. Scarlett, Cody J. Wenthur

**Affiliations:** ^1^School of Pharmacy, University of Wisconsin-Madison, Madison, WI, United States; ^2^Analytical Instrumentation Center, School of Pharmacy, University of Wisconsin-Madison, Madison, WI, United States; ^3^School of Pharmacy, Transdisciplinary Center for Research in Psychoactive Substances, University of Wisconsin-Madison, Madison, WI, United States

**Keywords:** psilocin, psilocybin, psilacetin, pharmacokinetic and pharmacodynamic parameters, prodrug

## Abstract

**Introduction:**

The use of the psychedelic compound psilocybin in conjunction with psychotherapy has shown promising results in the treatment of psychiatric disorders, though the underlying mechanisms supporting these effects remain unclear. Psilocybin is a Schedule I substance that is dephosphorylated *in vivo* to form an active metabolite, psilocin. Psilacetin, also known as O-acetylpsilocin or 4-acetoxy-N,N-dimethyltryptamine (4-AcO-DMT), is an unscheduled compound that has long been suggested as an alternative psilocin prodrug, though direct *in vivo* support for this hypothesis has thus far been lacking.

**Methods:**

This study employed liquid chromatography–tandem mass spectrometry (LC–MS/MS) to assess the time-course and plasma concentrations of psilocin following the intraperitoneal (IP) administration of psilacetin fumarate or psilocybin to male and female C57Bl6/J mice.

**Results:**

Direct comparisons of the time courses for psilocin exposure arising from psilocybin and psilacetin found that psilocybin led to 10–25% higher psilocin concentrations than psilacetin at 15-min post-injection. The half-life of psilocin remained approximately 30 min, irrespective of whether it came from psilocybin or psilacetin. Overall, the relative amount of psilocin exposure from psilacetin fumarate was found to be approximately 70% of that from psilocybin.

**Discussion:**

These findings provide the first direct support for the long-standing assumption in the field that psilacetin functions as a prodrug for psilocin *in vivo*. In addition, these results indicate that psilacetin fumarate results in lower peripheral psilocin exposure than psilocybin when dosed on an equimolar basis. Thoughtful substitution of psilocybin with psilacetin fumarate appears to be a viable approach for conducting mechanistic psychedelic research in C57Bl6/J mice.

## Introduction

### Comparison of psilocybin and psilacetin

**Figure 1:**
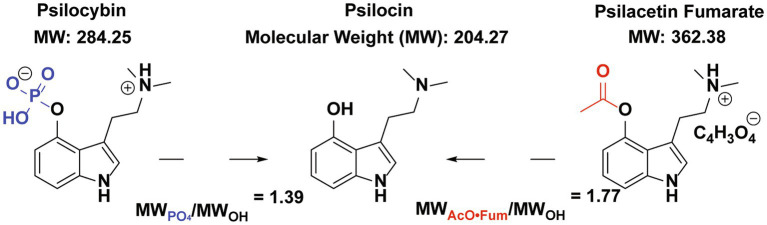
Psilocybin and psilacetin (in fumarate salt form) as prodrugs for Psilocin. The relevant protecting groups for psilocin’s hydroxyl group are shown in blue for psilocybin and red for psilacetin fumarate. Based on relative molecular weights, doses of 1.39 mg/kg of psilocin or 1.77 mg/kg of psilacetin fumarate by weight are needed to yield a 1 mg/kg equivalent dose of psilocin.

Psilocybin is a Schedule 1 compound being investigated for the treatment of major depressive disorder and other psychiatric conditions (1–10). Psilocybin is rapidly dephosphorylated in the body and is thought to predominantly act as a prodrug to deliver the active metabolite psilocin, another Schedule 1 substance (11, 12). In human studies, the pharmacokinetics and exposure of psilocin have been well validated *in vivo* after administering psilocybin in accordance with both fixed-dose and weight-based protocols (13–16). While psilocybin has the longest history of human consumption of any known psilocin prodrug, as this natural product found in *Psilocybe* mushrooms has been available since antiquity, it is not the only psilocin prodrug known (Figure 1) (1, 17–20).

Another notable example is the synthetic tryptamine psilacetin, also known as O-acetylpsilocin and 4-acetoxy-N,N-dimethyltryptamine (4-AcO-DMT). Psilacetin was first disclosed in a patent by Hofmann and Troxler in 1961, with an improved synthesis disclosed by Nichols and Frescas in 1999 (21, 22). Recently, both fumarate and hemifumarate crystalline forms of psilacetin have been isolated (23, 24). Although psilacetin induces psychedelic effects in humans, it is not presently included in any international drug schedules, including the UN 1971 Convention on Psychotropic Substances, which established a system for classifying controlled psychoactive drugs into four schedules based on their potential for abuse and therapeutic value (1, 25–27).

In recent years, there has been a growing interest in studying psilacetin as an alternative to psilocybin, for several reasons. First, due to its entirely synthetic nature, psilacetin offers researchers greater control over its production, distribution, and dosing compared to the variability inherent in extractions of psilocybin from naturally occurring psilocybin-containing mushrooms (21, 28–32). Even when comparing the synthetic production of psilacetin versus psilocybin, the production of psilacetin is notably simpler, with superior atom economy and fewer steps. This is primarily due to the difficulty encountered in the installation of the phosphate group of psilocybin (28, 30, 33). Additionally, substituting psilacetin for psilocin may reduce regulatory access barriers for researchers who do not hold a Schedule I DEA research license.

### Psilacetin usage, effects, and pharmacology

Investigation of psilacetin is also of significant public health relevance due to its recreational use. Synthetic tryptamines have been available on the designer drug market since at least the late 1990s (34), with psilacetin being a notable contributor to this marketplace. In Spain, the drug testing organization Energy Control has identified psilacetin as the most prevalent non-regulated tryptamine in samples submitted between 2006 and 2015 (35). Furthermore, the prevalence of psilacetin use has even been suggested to surpass that of psychedelic mushroom use in recent years (36). Individuals taking psilacetin describe its effects as comparable to those of psilocybin, yet without the adverse side effects associated with the use of whole *Psilocybe* mushrooms, such as nausea (36, 37). Nevertheless, there remains a paucity of academic studies concentrating on psilacetin (32) in comparison to the numerous research efforts on the pharmacological impacts and metabolism of psilocybin and psilocin (38–41).

While metabolism to psilocin has been suggested as the likely source of psilacetin’s psychoactivity, there remains ambiguity as to whether the parent drug itself exerts additional behaviorally meaningful pharmacologic effects of its own across species (1, 32). Some 4-acetoxy-N,N-dialkyltryptamines have been reported in humans to exhibit effects reminiscent of lysergic acid diethylamide (LSD) and have been suggested to have enhanced passive access to the brain due to the acetoxy group enhancing lipid solubility, thereby aiding in crossing the blood–brain barrier (33). The 5-HT_2A_ receptor is viewed as a significant target for psychoactive tryptamines such as psilacetin, as it is with other classical psychedelics such as psilocybin. *In vitro*, psilacetin’s receptor potency is approximately 10- to 20-fold lower than that of psilocin, though this difference has little apparent influence on HTR potency *in vivo* (30, 33). Psilacetin induced equivalent head twitch responses to psilocin on an equimolar basis: psilocin at 0.81 μmol/kg and psilacetin at 1.12 μmol/kg (33). Psilacetin has also demonstrated overlapping 95% confidence intervals with psilocybin regarding potency for inducing head twitch, hypolocomotion, and hypothermia in rodents, despite psilacetin having substantially higher affinity and potency at 5-HT_2a_ than psilocybin (30). Together, these results are consistent with psilacetin’s behavioral effects in animal models being predominantly driven by psilocin liberation *in vivo*.

### The present study

There is a standing call in the field for direct evidence of psilacetin’s *in vivo* conversion to psilocin to confirm its long-assumed prodrug status; evidence for this transformation has come only from *in vitro* studies to date (30, 33). In this study, we directly respond to this request. A liquid chromatography–tandem mass spectrometry method suitable for the quantitative analysis of psilocin concentrations is assessed for accuracy and applied to determine the plasma concentrations of liberated psilocin following the administration of psilacetin fumarate and psilocybin to mice.

## Materials and methods

### Animals and husbandry

All experimental procedures were approved by the University of Wisconsin – Madison Animal Care and Use Committee (IACUC) and completed in full accordance with Research Animal Resources and Compliance (RARC) guidelines. All 115 mice used in this study were acclimated to the University of Wisconsin vivarium conditions for at least 7 days prior to handling or experimentation. Food pellets (LabDiet) and water (Inno-Vive) were available *ad libitum*, unless otherwise noted. All C57Bl6/J mice used (male and female; 6–8 weeks old; The Jackson Laboratory, ME, USA) were housed in groups of three or four while under a 12 h artificial, reversed light/dark cycle. The room temperature remained constant between 22 and 24°C.

### Drugs

All controlled substances were handled by authorized users on Schedule I and Schedules II − V DEA research licenses and WI Special Use Authorizations held by Dr. Cody Wenthur. For *in vivo* injections, psilocybin powder (Usona Institute; Madison, WI; > 99% purity) was diluted in 0.9% sterile saline, then acidified to a pH of 1–2 with 1 M HCl, sonicated for 30–60 s, and brought to a pH of 6–7 using 1 M NaOH. Psilacetin fumarate (1:1) (Usona Institute; Madison, WI; >99% purity) was diluted in 0.9% sterile saline at pH 6–7. These materials were passed through a 0.2 μm filter and administered intraperitoneally (IP). All IP injections were given at a volume of 10 mL/kg. Chemical purity was assessed for all compounds using high-resolution LC–MS, and the fumarate anion 1:1 molar ratio for psilacetin fumarate was verified using ^1^H NMR. No mouse was given more than one injection of psilocybin or psilacetin fumarate or re-used following a washout period.

### Blood sample collection

Animals were briefly anesthetized with isoflurane (to prevent loss of righting reflex) prior to decapitation and trunk blood collection. Collections occurred at time points between 15 and 240 min after drug administration using EDTA-coated microcentrifuge tubes. Following collection, the samples were then centrifuged at 10,000 rpm (11,292 g) for 10 min at 4°C. The plasma fraction was separated and stored in the dark at −80°C until LC–MS/MS analysis.

### Liquid chromatography/tandem mass spectrometry (LC–MS/MS)

LC–MS/MS analysis and quantitation occurred in the Analytical Instrumentation Center (AIC) at the UW School of Pharmacy. For the preparation of analytical standards, psilocin (Usona Institute, Madison, WI; > 99% purity) was prepared in Optima LC–MS-grade methanol (Fisher Scientific, Hampton, NH). d_10_-Psilocin solution was purchased from Cerilliant (Round Rock, TX) for use as an internal standard (ITSD). Blank mouse plasma for preparing calibration curves and quality control samples (QCs) was purchased from Innovative Research (Novi, MI). All solvents for liquid chromatography were Optima LC/MS grade (Fisher Scientific, Hampton, NH). Additives for LC/MS analysis were purchased from Sigma Aldrich (St. Louis, MO).

Calibrators and QCs were prepared from stock methanol solutions of active pharmaceutical ingredients diluted to between 0.5 and 400 ng/mL in blank plasma. Samples, calibrators, and QCs were prepared for LC–MS/MS by protein precipitation and filtration using Waters Sirocco plates (Milford, MA) according to the manufacturer’s instructions. A precipitation mix containing the ISTD was prepared and aliquoted to a Sirocco plate mounted on a 96-well receiver. Samples, QCs, and calibrators were added to the plate, incubated for 2 min, and pushed through the plate using a positive pressure manifold (Waters, Milford, MA). Processed samples were then dried under nitrogen and resuspended in 100 μL of 98% A/2% B solvent prior to LC/MS/MS analysis.

Quantitative LC–MS/MS was performed using a Waters Acquity I-Class binary pump (Waters Corp., Milford MA) coupled to a Sciex QTRAP 5500 mass spectrometer (Sciex Corp., Framingham MA). Samples were separated on a Kinetex Core-Shell phenyl-hexyl 2.1 × 100 mm column (Phenomenex, Torrence, CA) using a 3-min gradient with a flow rate of 0.4 mL/min and a column temperature of 28°C. The initial conditions consisted of 95% solvent A (2.5 mM ammonium formate in water with 0.1% formic acid) and 5% solvent B (acetonitrile with 0.1% formic acid). The elution gradient began at 5% B, increased to 8.6% B over 1.8 min, and then quickly increased to 95% B in 0.15 min. This was held for 0.6 min, then decreased to 5% B in another 0.15 min, followed by a 0.25-min re-equilibration period. The column temperature was maintained at 28°C with a flow rate of 0.4 mL/min. All samples were injected in triplicate in randomized order, and the average of these injections was used for analysis. Blanks were injected between each calibrant, QC, or sample injection. For quantitation, the area under the curve of analyte peaks relative to ISTD peaks was modeled for each identified transition using a quadratic curve fit with 1/x^2^ weighting. Data was processed using MultiQuant software (Sciex, Framingham, MA). Any calibrator points differing by more than 15% from theoretical values were eliminated from the model.

### Pharmacokinetic calculations

The elimination rate (K_e_) for psilocin was determined by fitting a linear regression to the plots of the natural log of psilocin concentration over time for each tested condition, then averaging the values of the reported slopes. The elimination rate was reported as the inverse of this average slope. Half-life was calculated from this elimination rate using T_1/2_ = Ln (2)/K_e_.

Relative exposure of psilocin was calculated for each pairwise comparison between psilacetin (A) and psilocybin (B) doses using F = (AUC_A_/Dose A)/(AUC_B_/Dose B). Relative exposure was then reported from the average of the four pairwise dose comparisons.

### Statistical analysis

Statistical analyses were performed using GraphPad Prism, version 10 (San Diego, CA). All tests were run as two-tailed analyses, with a value of *p* of <0.05 as the threshold for significance. Post-hoc tests for ANOVA are reported with *p*-values corrected for multiple comparisons when follow-up tests were run to assess differences between specific conditions. Tests for outliers in biological data were run using ROUT at a 1% threshold, and one outlier was removed based on this threshold overall.

## Results

### Analytical methodology assessment

**Figure 2:**
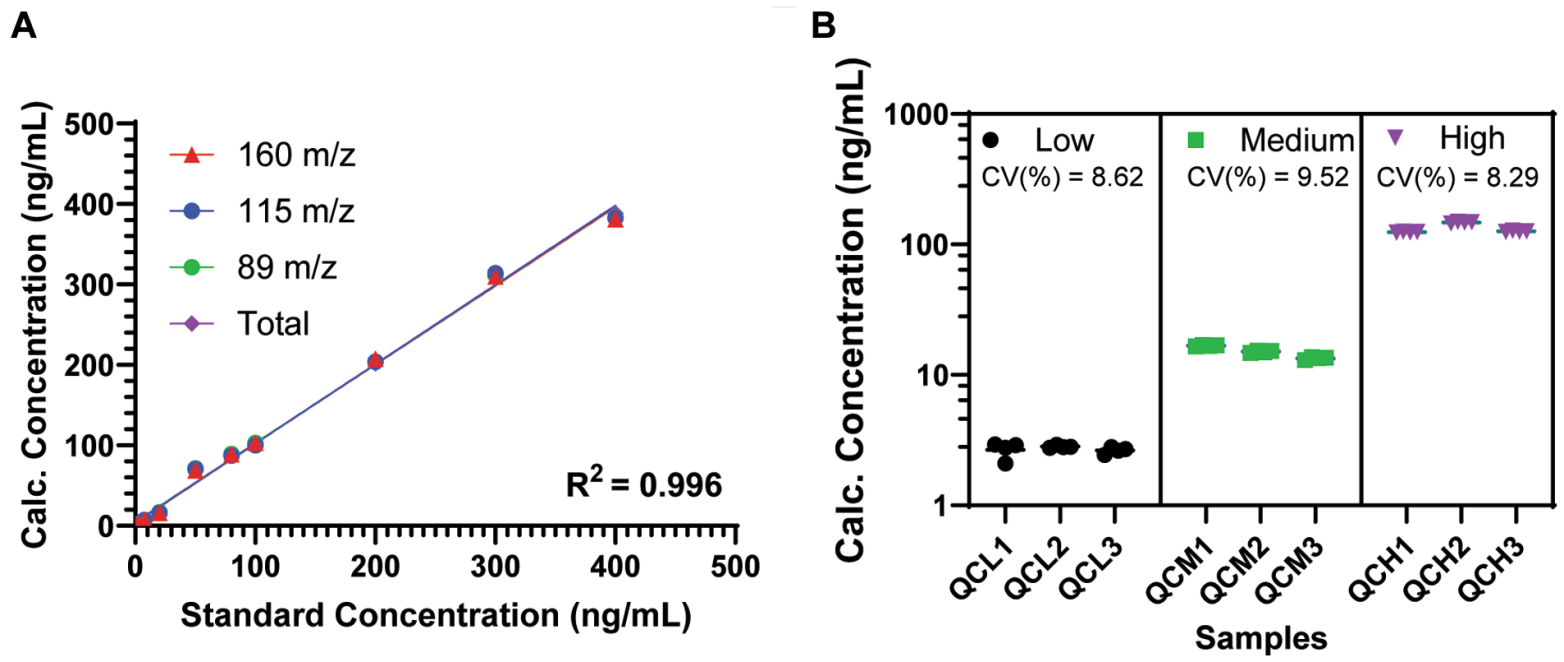
Performance of the tandem LC–MS/MS method for psilocin detection in mouse plasma. **(A)** Standard curves for observed psilocin transitions. R^2^ is shown as the average of all four curves (range, 0.9951–0.9968). **(B)** Quality control samples shows variation for multiple injections and across multiple samples at low, medium, and high concentrations. Coefficients of variation (CV%) are shown across all transitions, injections, and samples at each concentration. Data are shown as mean ± SEM.

The LC–MS/MS method used for the identification of psilocin in mouse plasma samples yielded three distinct transitions for psilocin at 205/160, 205/115, and 205/89 m/z. Curve fits for each independent transition, as well as a total fit weighted across the three transitions, yielded suitable standard curves for analytical application with R^2^ values >0.995 and coefficients of variation <10% across all QC samples, injections, and transitions (Figure 2). The total weighted curve fit was selected for use in the assessment of biological samples, as it had the highest R^2^ value overall at 0.9968.

### Verification of *in vivo* production of psilocin from psilacetin and psilocybin

**Figure 3:**
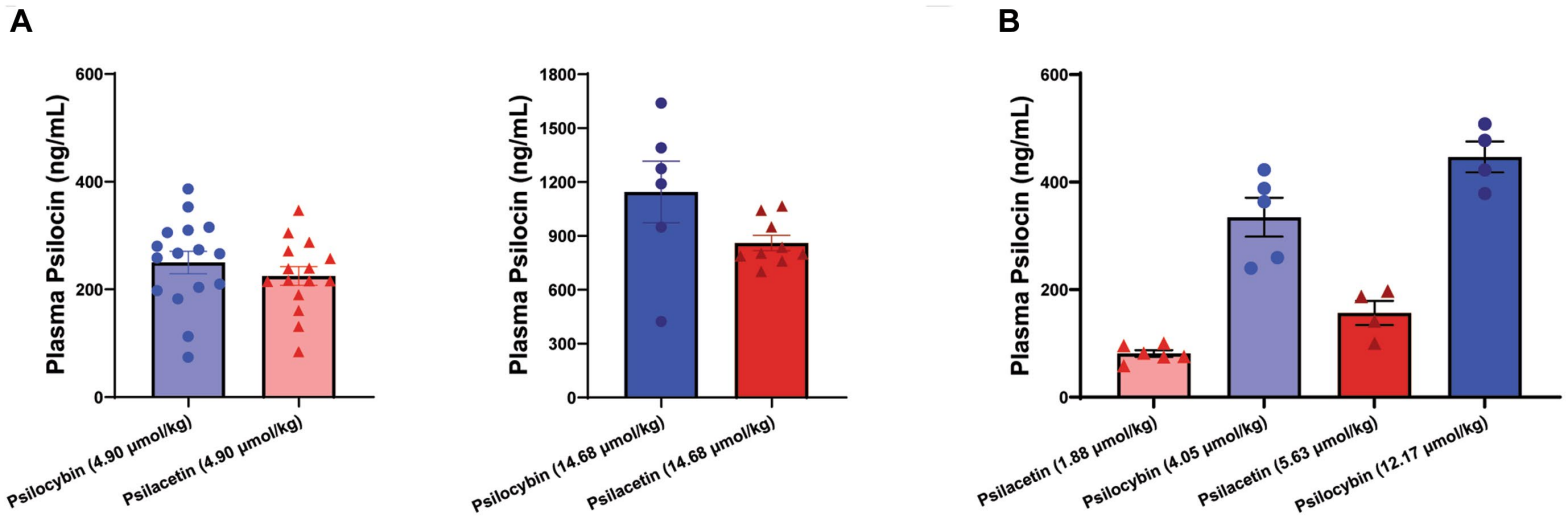
Metabolically derived psilocin is rapidly detectable following psilocybin and psilacetin fumarate injections in mice. Psilocin concentrations were detected in plasma 15 min after intraperitoneal injection. **(A)** Matched equimolar doses equivalent to either 1 mg/kg psilocin (4.90 μmol/kg) or 3 mg/kg psilocin (14.68 μmol/kg). One outlier was removed for psilacetin at 14.68 μmol/kg (ROUT, 1%). **(B)** Interleaved doses of psilacetin fumarate (1.88 μmol/kg; 5.63 μmol/kg) and psilocybin (4.05 μmol/kg; 12.17 μmol/kg) across an escalating range. Data are shown as mean ± SEM.

This LC–MS/MS approach was used to assess plasma samples collected from animals treated with psilocybin and psilacetin fumarate at 15 min after administration (Figure 3). To look at relative concentrations of psilocin liberated into the plasma, the doses were administered on an equimolar basis and were selected to be equivalent to the administration of either 1 mg/kg of psilocin or 3 mg/kg of psilocin. Notably, micromolar concentrations of psilocin were found in the plasma of animals treated with psilacetin at both doses, indicating robust metabolic transformation *in vivo*. The psilocin concentration resulting from the 1 mg/kg equivalent dose of psilacetin was 225 ng/mL, which was 90% of that from psilocybin (250 ng/mL). For the 3 mg/kg equivalent dose, the psilacetin concentration was 860 ng/mL, or 75% of that from psilocybin (1,145 ng/mL). These psilocin concentrations were not found to be significantly different between prodrugs at these sample sizes (Student’s t-test: 1 mg/kg, *p* = 0.37; 3 mg/kg, *p* = 0.08).

The trend showing a lower fraction of psilocin exposure following psilacetin exposure than following psilocybin exposure was supported in a second experiment using doses of psilacetin fumarate and psilocybin that were interleaved across an escalating dose scale. As expected, these doses resulted in significantly different concentrations of plasma psilocin overall (ANOVA, *F* = 45.74, *p* < 0.0001). However, there was not a smoothly increasing concentration of psilocin across escalating doses, as would be expected if psilocin exposure from both prodrugs was equal. Instead, the 5.63 μmol/kg dose of psilacetin fumarate had a lower psilocin concentration than that from the 4.90 μmol/kg dose of psilocybin.

### Time course and magnitude of psilocin exposure from psilacetin and psilocybin

**Figure 4:**
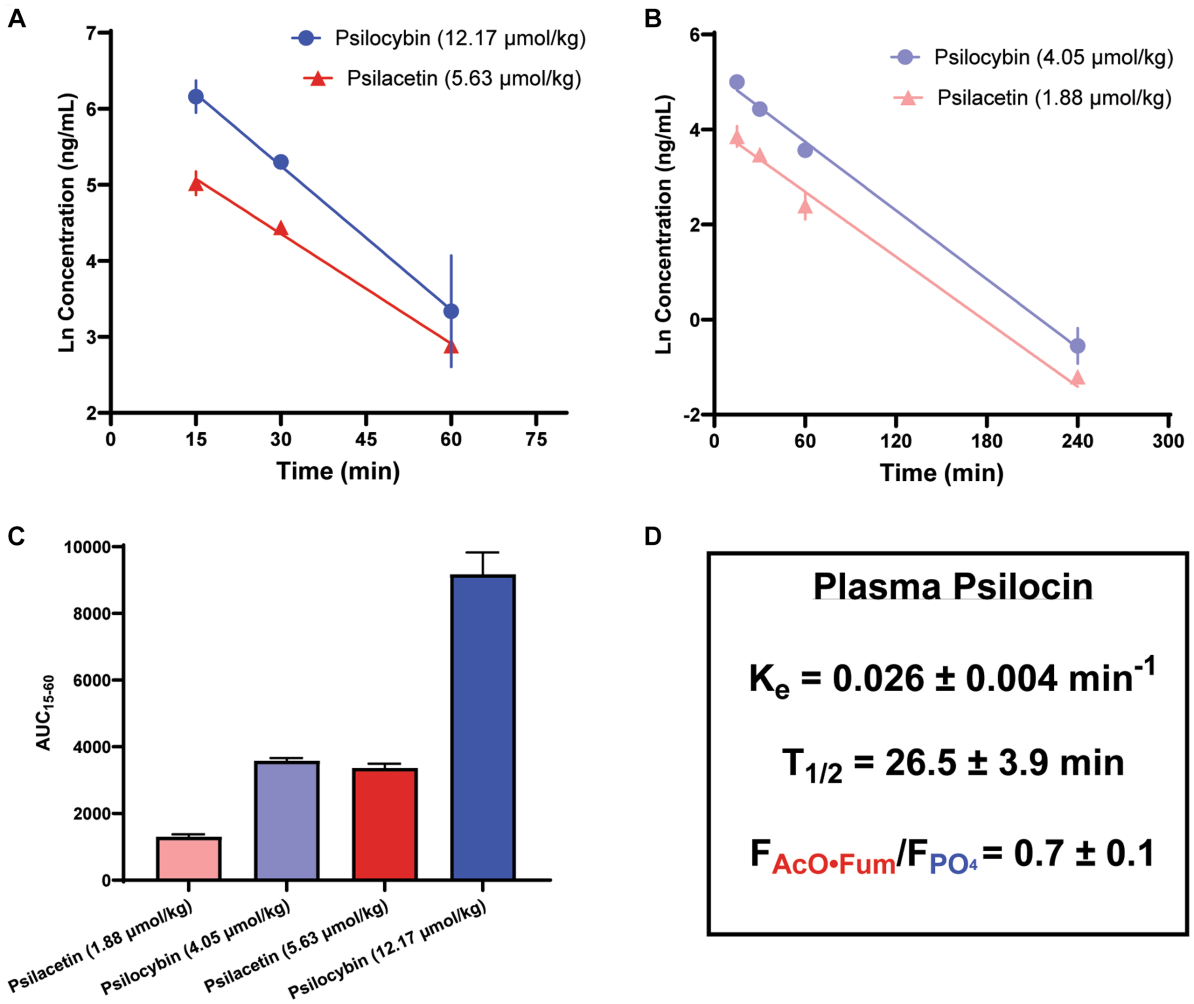
Metabolism of psilacetin fumarate in mice yields a lower psilocin plasma exposure as compared to the metabolism of psilocybin. Psilocin plasma concentration time courses from **(A)** doses of psilocybin (12.17 μmol/kg; *n* = 12, 4/timepoint) and psilacetin fumarate (5.68 μmol/kg; *n* = 9, 3/timepoint) between 15 and 60 min after intraperitoneal injection and **(B)** doses of psilocybin (4.05 μmol/kg; *n* = 9, 3/timepoint) and psilacetin fumarate (1.88 μmol/kg *n* = 9, 3/timepoint) between 15 and 240 min after intraperitoneal injection. Lines show best-fit linear regressions for a first-order elimination model. **(C)** Comparison of areas under the curves from 15 to 60 min following intraperitoneal injection for all doses in panels **(A,B)**. **(D)** Elimination rate and half-life of liberated psilocin, as well as relative bioavailability of psilocin from psilacetin fumarate in comparison to psilocybin. Data are shown as mean ± SEM.

Given the relatively smaller sample size used and higher variability observed for the single-time point dose escalation experiment, a follow-up assessment of psilocin concentrations over time was also undertaken to further illuminate the full profiles of psilocybin and psilacetin fumarate as prodrugs. This analysis was undertaken using a separate cohort of animals from the single-time point experiments (Figure 4). The relative plasma profiles of psilocin liberated from either psilacetin fumarate or psilocybin demonstrated dose-dependency between 15 and 240 min that was consistent with the observations at 15 min. The elimination of psilocin liberated from either source was observed to adhere to first-order kinetics across doses. The terminal elimination rate was found to be 0.026 min^−1^ (range, 0.020–0.038 min^1^). This corresponds to a psilocin half-life of approximately one-half hour, aligning with previously reported results for the clearance of this molecule (42). Plasma psilocin concentrations fell to near or below the lower limit of quantitation for the LC/MS/MS assay (0.1 ng/mL) by 4 h after administration, or approximately 6 half-lives.

Notably, in an observation consistent with the single-time point experiments, psilacetin fumarate generated lower levels of plasma exposure to psilocin than psilocybin on an equimolar basis. When relative psilocin bioavailability was calculated using dose-corrected areas under the curves (AUCs) between 15 and 60 min, there were significant differences in AUCs across all groups (ANOVA, *F* = 107.6, *p* < 0.0001), and both drugs also independently demonstrated their own dose-dependent exposure trends (Sidak’s, psilacetin, *p* = 0.0002; psilocybin, *p* < 0.0001). However, psilacetin was found to yield only between 67 and 89% as much psilocin exposure in comparison to psilocybin. On average, across all possible pairwise dose comparisons, psilacetin resulted in approximately 30% less psilocin exposure than psilocybin on an equimolar basis. This is effectively demonstrated by comparing the profiles from the 5.63 μmol/kg dose of psilacetin fumarate and the 4.05 μmol/kg dose of psilocybin—these two conditions generated nearly identical psilocin exposure (Sidak’s, *p* = 0.95) despite the psilocybin dose being approximately 30% smaller than the psilacetin dose in terms of molar equivalents.

## Discussion

### Summary of the findings

The results of these experiments demonstrate that psilacetin fumarate acts as a prodrug for psilocin in both male and female C57Bl6/J mice. There was no sexual dimorphism in the production of psilocin from either psilacetin or psilocybin. While the *in vivo* action of psilacetin as a psilocin prodrug has long been hypothesized, this is the first formal, publicly available pharmacokinetic report validating this status *in vivo* of which we are aware.

### Theoretical and practical implications

This validation has important implications for pre-clinical psychedelic research programs. Most notably, regular substitution of psilacetin fumarate as an unscheduled (35) alternative to psilocybin in pre-clinical studies may enable broader access and more rapid progress on mechanistic questions surrounding psilocin’s effects. For clinical studies using human participants, there is less likelihood of accelerating progress through substitution alone, given the additional regulatory considerations involved. However, there may be other compensatory benefits to the pursuit of psilacetin or other novel psilocin prodrug strategies as alternatives to psilocybin for human research studies, such as the possibility of reduced ethical, legal, and sustainability concerns by avoiding the commercialization of a natural product with a long documented history of sacramental use by indigenous peoples (18, 43, 44).

### Limitations and future directions

There are several limitations to this study worth noting when considering psilacetin fumarate substitution for psilocybin in C57Bl6/J mice. First, while these data support the liberation of psilocin as an active metabolite that contributes to the actions of psilacetin, they do not address the relevant intrinsic pharmacologic activity of psilacetin, which may occur alongside psilocin. Second, these studies were limited to plasma, and central nervous system exposure may be different. Together, these factors mean that pharmacodynamically equivalent doses are not likely to be the same as the peripherally pharmacokinetically equivalent doses noted here. Furthermore, this effort used the fumarate, rather than hemifumarate, crystalline form—if using the hemifumarate form, equimolar dosage adjustments will be required to account for the half-weight of fumarate complexed with each psilacetin molecule. Finally, while this study aimed to explore dose ranges (0–5 mg/kg) for psilocybin and psilacetin that are commonly employed in pre-clinical studies, the resulting plasma concentrations observed (100–1,200 ng/mL) are significantly larger than those seen in human pharmacokinetic studies of psilocybin (5–50 ng/mL). This notable difference means that attempts to translate relative exposure outcomes across species are likely premature.

## Conclusion

In summary, the results of the experiments reported here provide direct evidence to validate the long-standing assumption that psilacetin acts as a psilocin prodrug *in vivo*. They also provide initial evidence suggesting that psilacetin fumarate leads to a quantifiably lower psilocin peripheral exposure as compared to psilocybin on an equimolar basis. Together, these findings provide an empirical basis for pre-clinical investigators to thoughtfully substitute the unscheduled compound psilacetin for the Schedule 1 compound psilocybin as a pharmacokinetically reasonable means to address a significant regulatory barrier to entry for new scientists interested in contributing to the growing field of psychedelic studies.

## Data availability statement

The original contributions presented in the study are publicly available. This data can be found here: https://figshare.com/articles/dataset/RawData_TandemLCMS_PsilocybinPsilacetin_Psilocin_PlasmaConcentrations/24434725.

## Ethics statement

The animal study was approved by University of Wisconsin – Madison Animal Care and Use Committee. The study was conducted in accordance with the local legislation and institutional requirements.

## Author contributions

NJ: Conceptualization, Formal analysis, Investigation, Methodology, Visualization, Writing – original draft, Writing – review & editing. LW: Formal analysis, Investigation, Writing – review & editing. MP: Formal analysis, Investigation, Methodology, Writing – review & editing. CS: Formal analysis, Investigation, Methodology, Supervision, Writing – review & editing. CW: Conceptualization, Data curation, Formal analysis, Funding acquisition, Project administration, Supervision, Visualization, Writing – original draft, Writing – review & editing.
